# Supplementary material for: Nr4a1 and Nr4a3 redundantly control clonal deletion and contribute to an anergy-like transcriptome in auto-reactive thymocytes to impose tolerance in mice
Source: Nat Commun. 2025 Jan 17;16:784. doi: 10.1038/s41467-025-55839-5 (PMC11742425; doi:10.1038/s41467-025-55839-5)
Supplement: Supplementary file 1 — Supplementary Information [file 41467_2025_55839_MOESM1_ESM.pdf]

Inventory of Supplementary Information

Title: Transcriptional control of central T cell tolerance by NR4A family nuclear receptors
First author: Hailyn V. Nielsen
Corresponding authors: Byron B. Au-Yeung and Julie Zikherman

Supplementary Figure 1. *Nr4a1, 2, 3* expression pattern (related to Main Fig 1)

Supplementary Figure 2. Polyclonal DKO chimeras (related to Main Fig 1)

Supplementary Figure 3. *Nr4a1* and *Nr4a3* mediate negative selection in OTII/RIPmOVA model (related to Main Fig 2)

Supplementary Figure 4. *Nr4a1* and *Nr4a3* are required for *Bcl2l11*/BIM upregulation by mTEC Ag (related to Main Fig 3)

Supplementary Figure 5. Transcriptional program induced in thymocytes by self-Ag (related to Main Fig 4)

Supplementary Figure 6. Nr4a-dependent transcriptional program induced in thymocytes by self-Ag (related to Main Fig 4)

Supplementary Figure 7. Transcriptional program induced by Nr4a family in thymus and periphery (related to Main Fig 4)

Supplementary Figure 8. RTE exhibit imprint corresponding to thymic Ag encounter (related to Main Fig 5, 6)

Supplementary Figure 9. DKO thymocytes acquire an anergic program that persists in the periphery (related to Main Fig 7, 8)

Supplementary Figure 10. *Nr4a1* and *Nr4a3* are required for negative selection by tissue-specific Ag

Supplementary Figure 11. Model

Supp Figure 1. *Nr4a1, 2, 3* expression pattern (related to Fig 1)

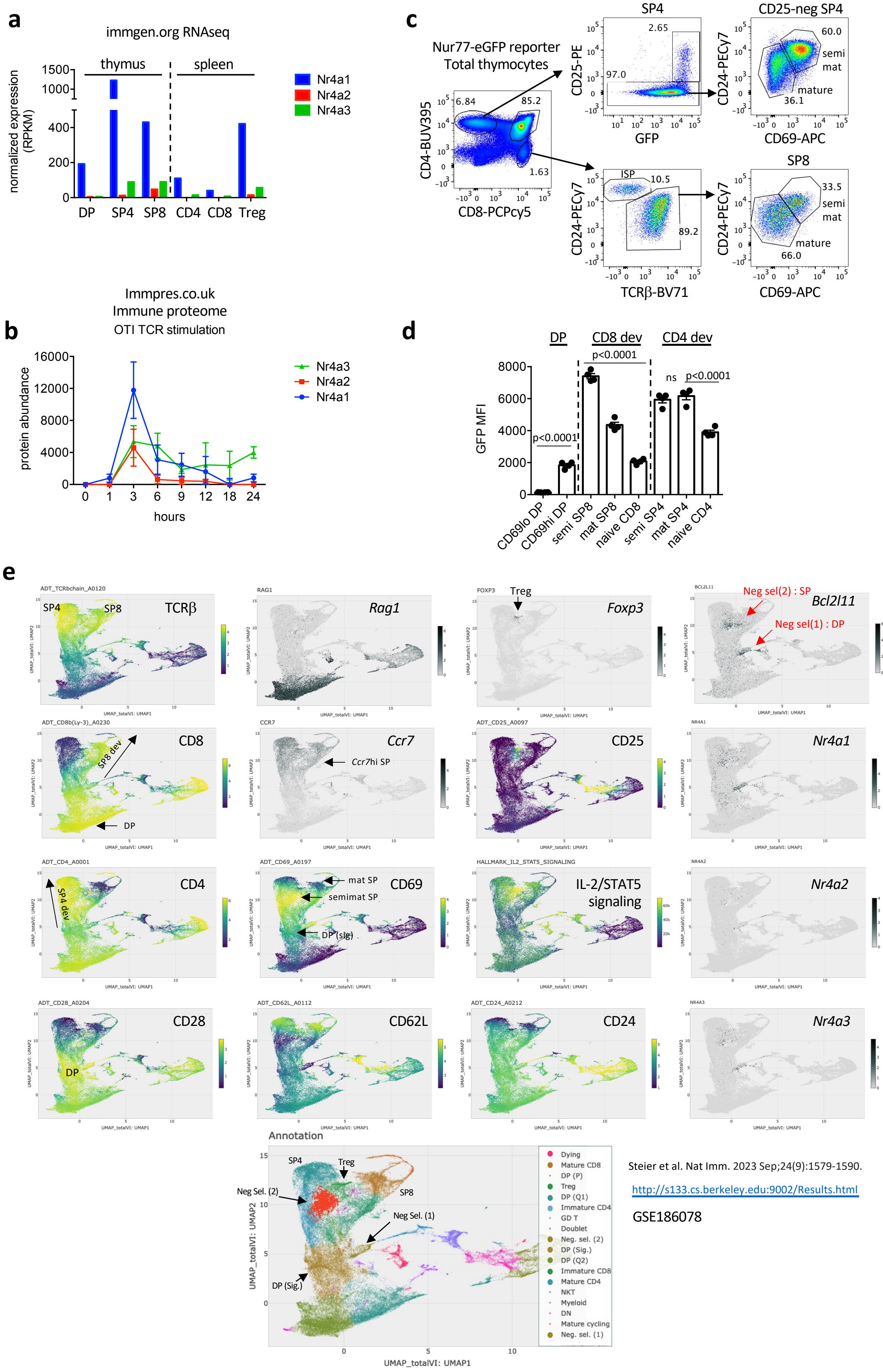

Supp Figure 1. Related to Main Figure 1

a. RPKM from public RNAseq data sets are plotted (immgen.org) depicting Nr4a1, 2, 3 transcript abundance among T cell lineage subsets in thymus and periphery.

b. mean +/- SD values from immune proteome database (Immpres.co.uk) are plotted depicting mass spec abundance of Nr4a1/2/3 protein products in TCR stimulated CD8 T cells across a time course.

c. Gating scheme depicts identification of DP and SP subsets among Nur77-eGFP reporter thymocytes.

d. Quantification of GFP MFI among Nur77-eGFP reporter thymic subsets as gated in c, in 4 biological replicates +/- SEM

e. Selected protein and transcript UMAP data from publicly available VISION thymus CITEseq interface (http://s133.cs.berkeley.edu:9002/Results.html; Steier et al. Nat Imm. 2023 Sep;24(9):1579-1590.)

Statistical tests:

d. one way ANOVA with Holm-Sidak correction for multiple pre-selected comparisons. No matching or pairing.

Data in c, d representative of 4 biological replicates

Source data are provided as a Source Data file.

Supp Figure 2. Polyclonal DKO chimeras (related to Fig 1)

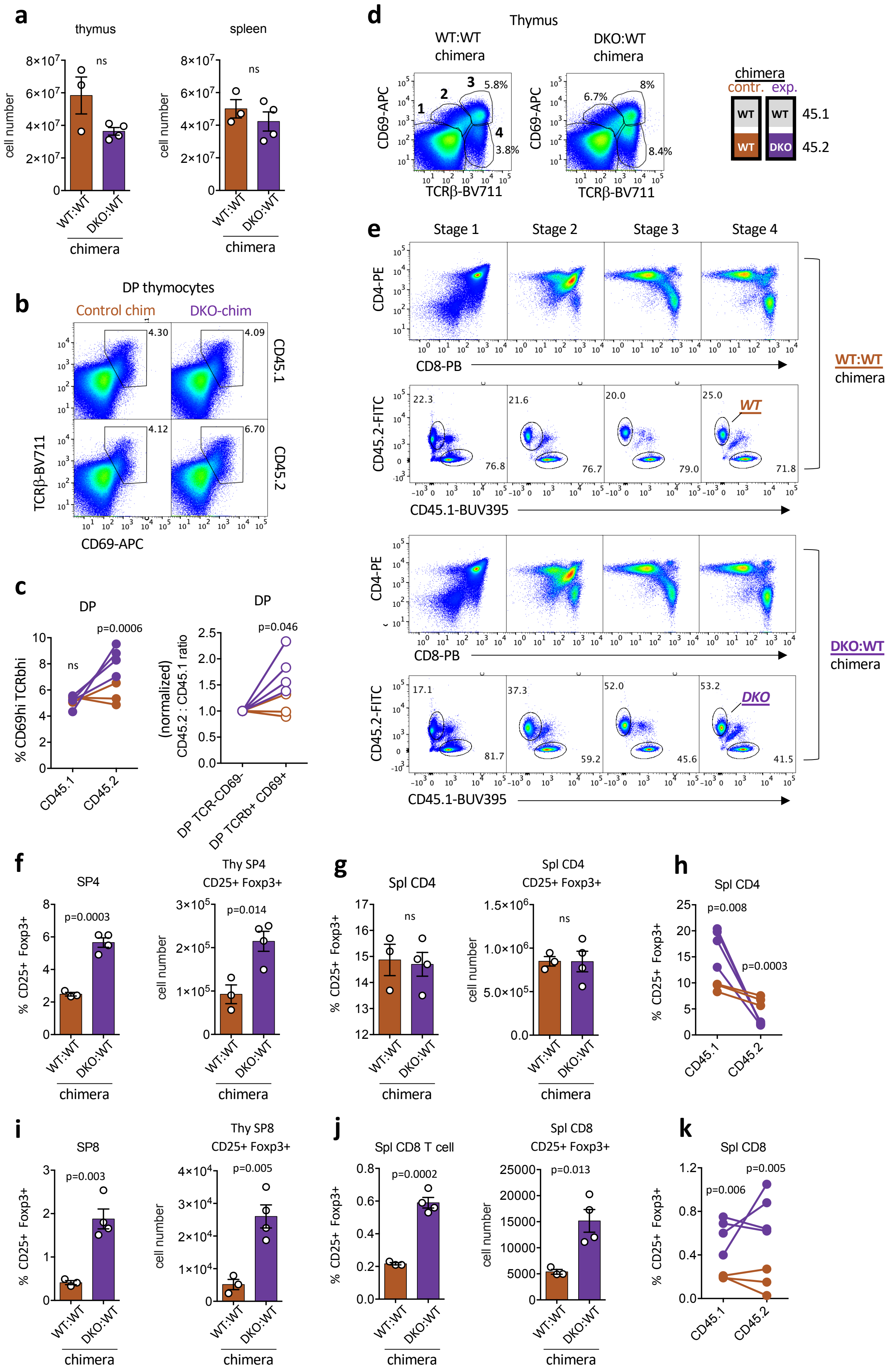

Supp Figure 2. Related to Main Figure 1

a. Graphs depict absolute cell number in thymus or spleen of polyclonal chimeras (see schematic Fig 1c) in biological replicates +/- SEM

b, c. Representative plots depict DP thymocytes of each donor genotype in polyclonal control and experimental chimeras (schematic in Fig 1c) stained to identify pre- and post-selection gates defined by CD69 and TCRβ expression. Graphs show quantification of gates (left) or ratio of donor genotypes (normalized to pre-selection DP) among these gates for biological replicates.

d, e. Representative plots (e) depict co-receptor agnostic gating to identify sequential stages of thymocyte development 1-4. In (e) thymic stages 1-4 as gated in (d) are shown for WT:WT (top) and DKO:WT (bottom) polyclonal chimeras (see schematic 1c). Both coreceptor expression (top) and CD45.1/CD45.2 donor genotypes (bottom) are depicted for each stage.

f, g. Graphs depict total % and absolute number of Foxp3+ CD4+ Treg (irrespective of donor genotype) from thymus and spleen of experimental or control polyclonal chimeras (schematic in Fig 1c) in biological replicates +/- SEM.

h. Graph depicts % Foxp3+ CD4+ Treg of each donor genotype in polyclonal chimeras. Lines depict donors from the same chimera.

i, j. Graphs depict total % and absolute number of Foxp3+ CD8+ cells (irrespective of donor genotype) from thymus and spleen of experimental or control polyclonal chimeras (schematic in Fig 1c) in biological replicates +/- SEM.

k. Graph depicts % Foxp3+ CD8+ cells of each donor genotype in polyclonal chimeras. Lines depict donors from the same chimera.

<u>Statistical tests</u> :

a, c, f-k, unpaired two-tailed parametric t-test, assume equal SD

Graphs in this fig a, c, f-k depict N=3 control chimeras and N=4 polyclonal chimeras (biological replicates) and are representative of 3 independent sets of chimeras.

Source data are provided as a Source Data file.

Supp Figure 3. *Nr4a1* and *Nr4a3* mediate negative selection in OTII/RIPmOVA model (related to Fig 2)

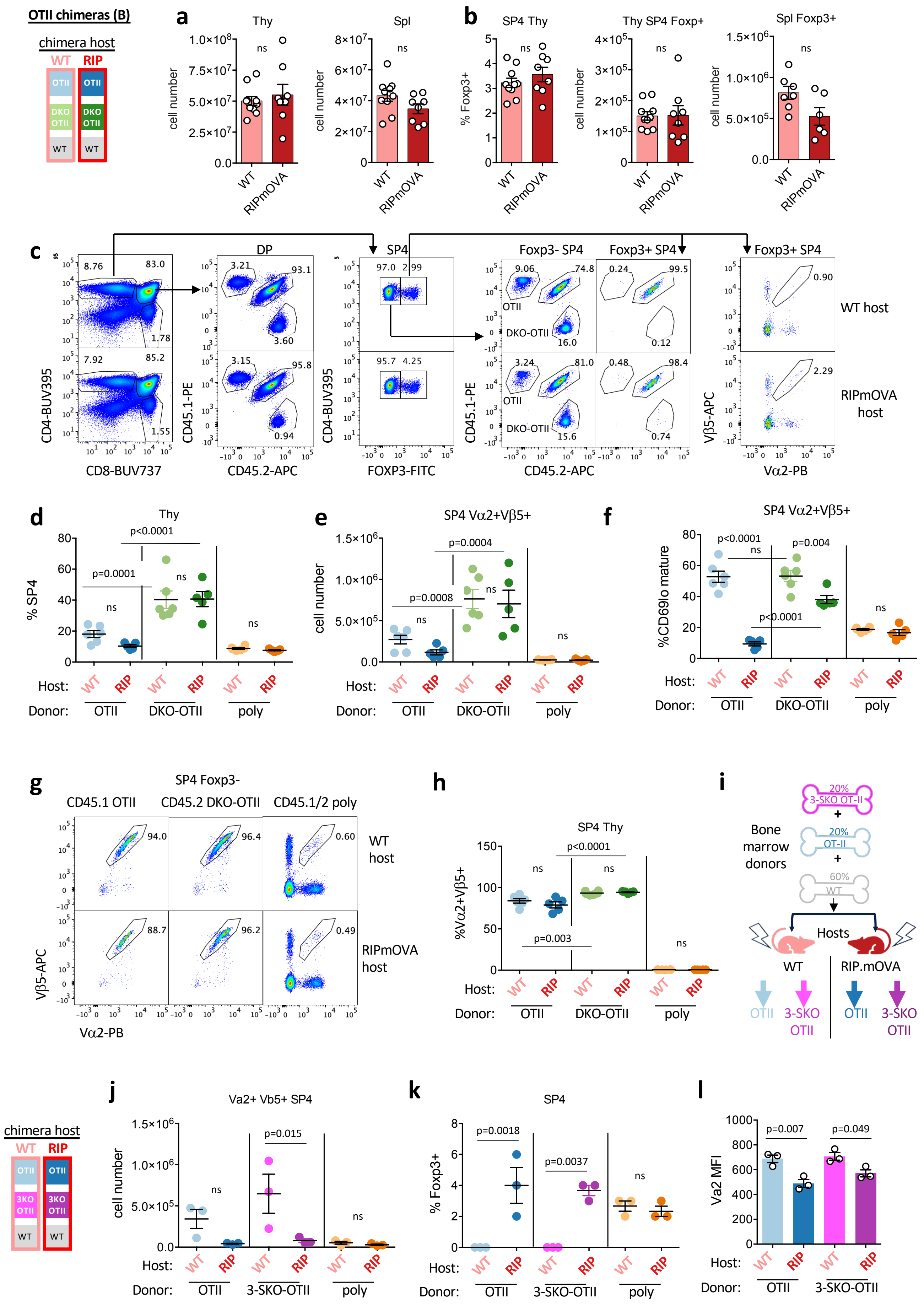

Supp Figure 3. Related to Main Figure 2

a. Graphs depict absolute cell number in thymus or spleen of RIPmOVA and WT host chimeras (see schematic Fig 2a) in biological replicates +/- SEM

b. Graphs depict relative and absolute number of Foxp3+ Treg in thymus and periphery of RIPmOVA and WT host chimeras (see schematic Fig 3a) in biological replicates +/- SEM

c. Representative gating scheme depicts donor genotypes among Foxp3- and Foxp3+ SP4 thymocytes as well as surface TCR chain expression among Foxp3+ SP4.

d, e. Graph depicts % (d) and absolute number (e) of Vα2+Vβ5+ SP4 populations from donor genotypes from WT host and RIPmOVA host chimeras (see Fig 3a schematic)

f. Graph depicts % of CD69lo cells +/- SEM (based on bimodal CD69 distribution in top panel of 2h).

g, h. Representative plots depict Foxp3- SP4 thymocytes of each donor genotype from WT host and RIPmOVA host chimeras (see Fig 3a schematic) stained and gated to detect Vα2+Vβ5+ cells. Graph (h) depicts quantification of gating in (g) for biological replicates +/- SEM.

i. Schematic depicts radiation chimera design analogous to design of Main Fig 2a in order to assess *Nr4a3-/-* single knockout (SKO) cells in OTII/RIPmOVA model. Analysis of these cjhimeras is presented in panels j, k, l below as well as Supp Fig 4c, d.

j, k. Graph depicts absolute number of Vα2+Vβ5+ SP4 populations (j) or % Foxp3+ cells among SP4 (k) from donor genotypes from WT host and RIPmOVA host chimeras (see chimera schematic i above). Gating as per chimeras depicted in Main Fig 2a.

l. Graph depicts surface expression MFI of TCR Vα2 among Vα2+Vβ5+ SP4 thymocytes of each OTII donor genotype in chimeras depicted in panel i.

<u>Statistical tests :</u>

a, b, Unpaired 2-tailed parametric t test, assume equal SD.

d,f, k,l, one way ANOVA with Tukey's multiple hypothesis test.

e, h, j, one way ANOVA with pre-specified comparisons corrected for by Sidak test

a, b plot data from N=10 WT and N=8 RIPmOVA chimeras (biological replicates) as schematized in Fig 2a except for spl foxp3 graph : N=7 WT and N=6 RIPmOVA chimeras.

d-f, h plot data from N=6 WT and N=5 RIPmOVA chimeras (biological replicates) as schematized in Fig 2a. j-l plot data from N=3 WT and N=3 RIPmOVA chimeras (biological replicates) as schematized in i. Source data are provided as a Source Data file.

Supp Figure 4. *Nr4a1* and *Nr4a3* are required for *Bcl2l11*/BIM upregulation by mTEC Ag (related to Fig 3)

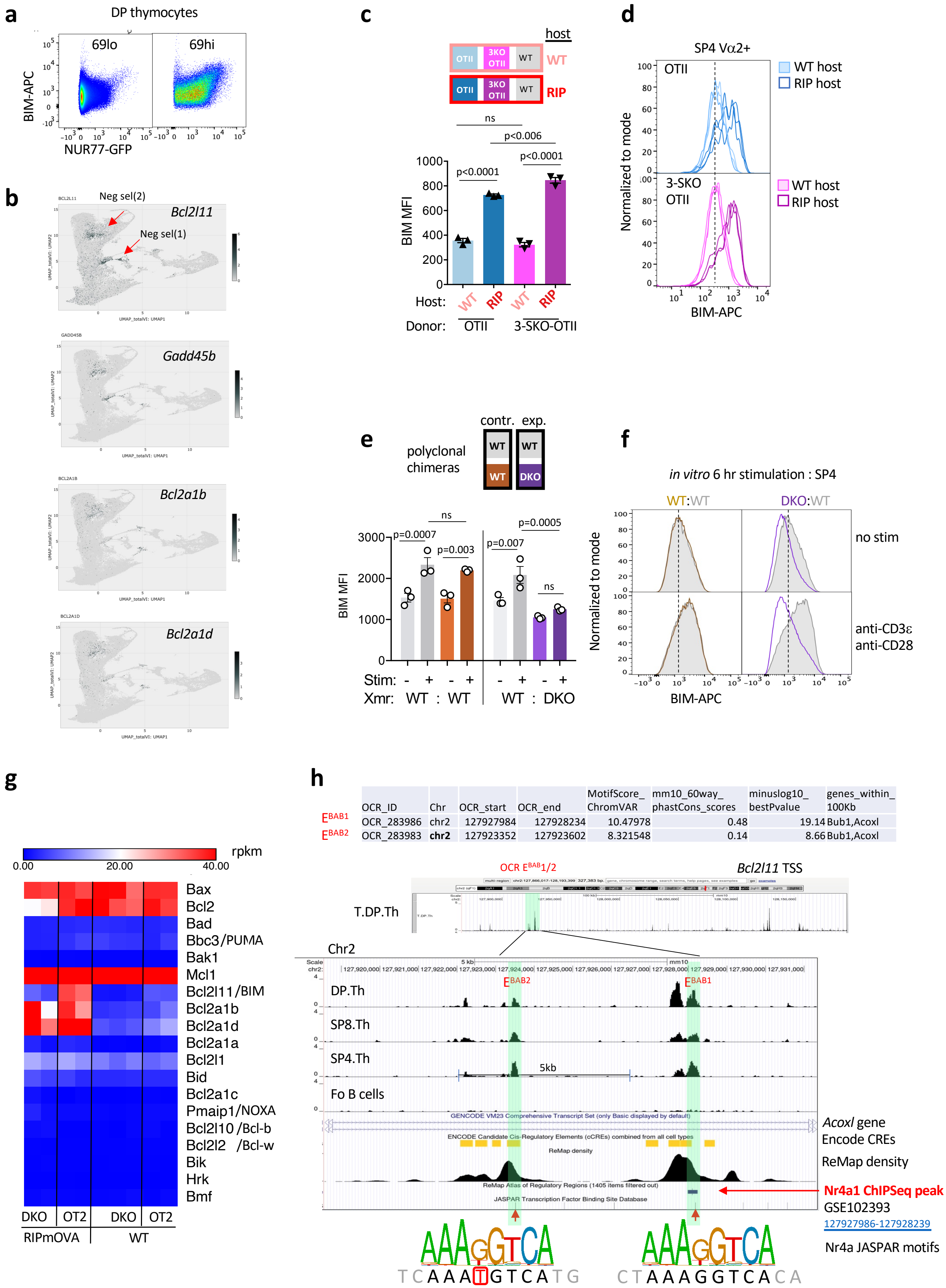

	OCR_ID	Chr	OCR_start	OCR_end	MotifScore_ChromVAR	mm10_60way_phastCons_scores	minuslog10_bestPvalue	genes_within_100Kb
EBAB1	OCR_283986	chr2	127927984	127928234	10.47978	0.48	19.14	Bub1,Acoxl
EBAB2	OCR_283983	chr2	127923352	127923602	8.321548	0.14	8.66	Bub1,Acoxl

Supp Figure 4. Related to Main Figure 3

a. DP thymocytes as described in Fig 3b are gated to identify pre-selection CD69lo and post-selection CD69hi subsets. Representative plots depict BIM and Nur77-GFP expression.

b. Selected transcript UMAP data from publicly available VISION thymus CITEseq interface (http://s133.cs.berkeley.edu:9002/Results.html; Steier et al. Nat Imm. 2023 Sep;24(9):1579-1590.)

c, d. Quantification and histograms depict ex vivo BIM staining of SP4 thymocytes from Nr4a3-/- SKO OTII chimera (see schematic Supp 3i and analysis Supp 3j-l). Experiment performed as in Main Fig 3h, i. 3 biological replicates are shown as overlaid histograms and plotted.

e, f. Quantification and representative histograms depict in vitro stimulation of thymocytes from DKO polyclonal chimeras (schematized in Main Fig 1c). Thymocytes from chimeras were stimulated with 10μg/ml 2c11 + 2μg/ml anti-CD28 for 6hr. Cells were then stained to detect thymic subsets with CD4 and CD8, congenic markers, and ic staining for BIM. 3 biological replicates are plotted in e.

g. Heatmap depicts absolute rpkm values for pro- and anti-apoptotic Bcl2 family members from RNAseq data set schematized in Fig 2a and described in Fig 4a (Supplementary Data 1a)

h. Genomic coordinates of E^{BAB1} and E^{BAB2} OCRs; UCSC browser tracks depict ATACseq peaks from public immgen.org as shown in Fig 3l but zoomed in.

Statistical tests:

c, one way ANOVA with Tukey's multiple hypothesis test.

e, two-way ANOVA with Tukey's multiple hypothesis test.

Source data are provided as a Source Data file.

Supp Figure 5. Transcriptional program induced in thymocytes by self-Ag (related to Fig 4)

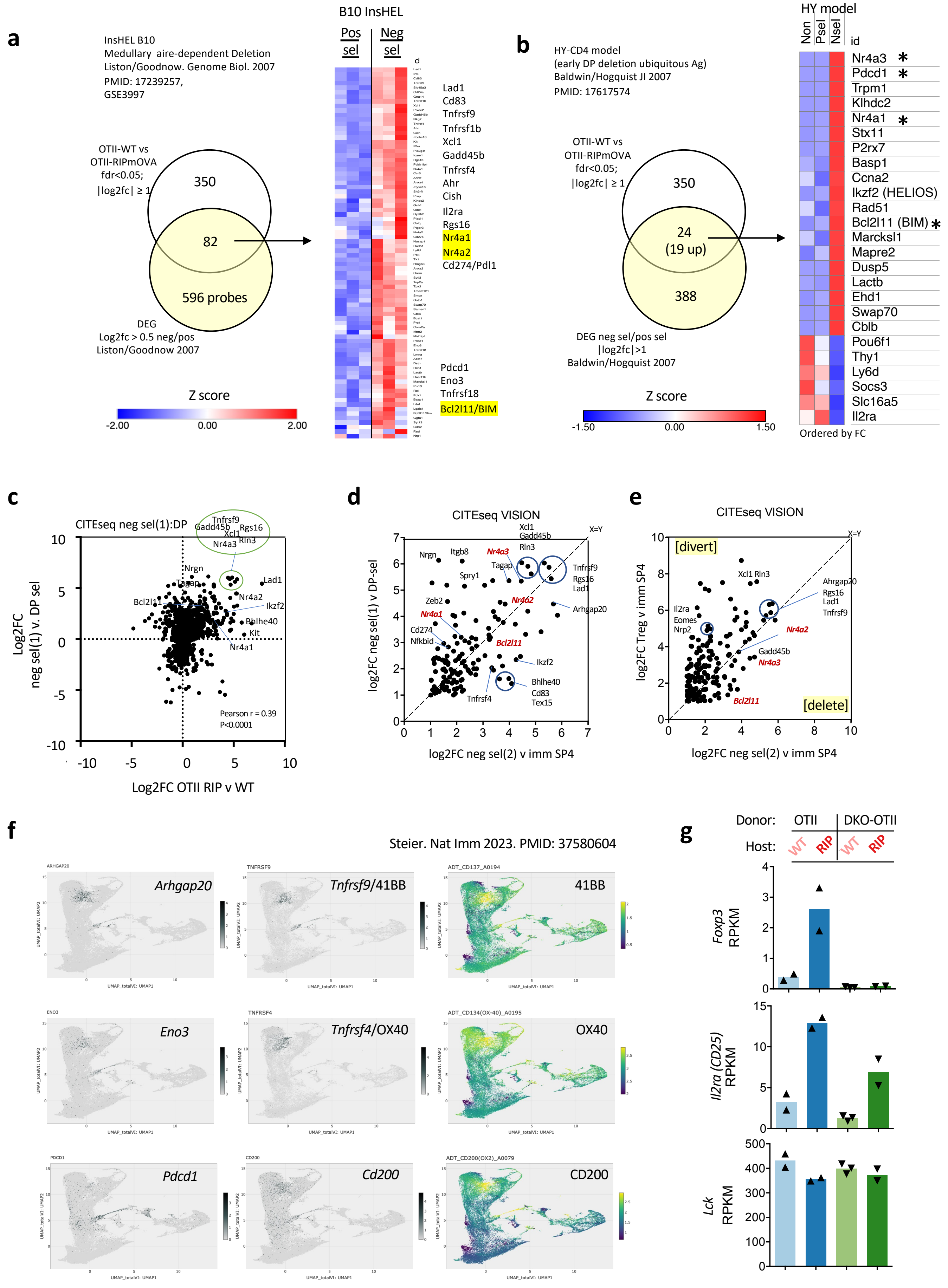

Supp Figure 5. Related to Main Figure 4

a. Venn diagram depicts overlap between OTII-RIPmOVA DEG (N=350 as in Fig 5a) and InsHEL model (PMID: 17239257, GSE3997). Heatmap depicts overlapping gene expression in InsHEL model. see also Supplementary Data 2a.

b. Venn diagram depicts overlap between OTII-RIPmOVA DEG as above and HY-CD4 model (PMID: 17617574). Heatmap depicts overlapping gene expression in HY-CD4 model. see also Supplementary Data 2b

c. FC FC plot as in Fig 5b except CITEseq comparator depict Log2FC Neg sel (1) cluster / signaled DP. Pearson correlation coefficient is quantified. See also Supplementary Data 2c.

d, e. FC FC plots as in c above except (d) comparing Neg Sel clusters 1, 2 to non-deleting closest comparitors (signaled DP and imm SP4 respectively), or (e) comparing Treg or Neg Sel 2 clusters each to imm SP4 from CITEseq VISION dataset. Plots include only DEG log2FC>1 AND fdr < 0.05. See also Supplementary Data 2d.

f. Selected protein and transcript UMAP data from publicly available VISION thymus CITEseq interface (http://s133.cs.berkeley.edu:9002/Results.html; Steier et al. Nat Imm. 2023 Sep;24(9):1579-1590.)

g. Graphs depict RPKM for *Foxp3, Il2ra*, and *Lck* in CD69hi Va2+ SP4 thymocytes of either WT-OTII or DKO-OTII donor genotypes from chimeras depicted in Main Fig 2a. RNAseq was performed on biological replicates (Supplementary Data 1a depicts p value and fdr for all pairwise comparisons across sample types, GSE235101).

Supp Figure 6. Nr4a-dependent transcriptional program induced in thymocytes by self-Ag (related to Fig 4)

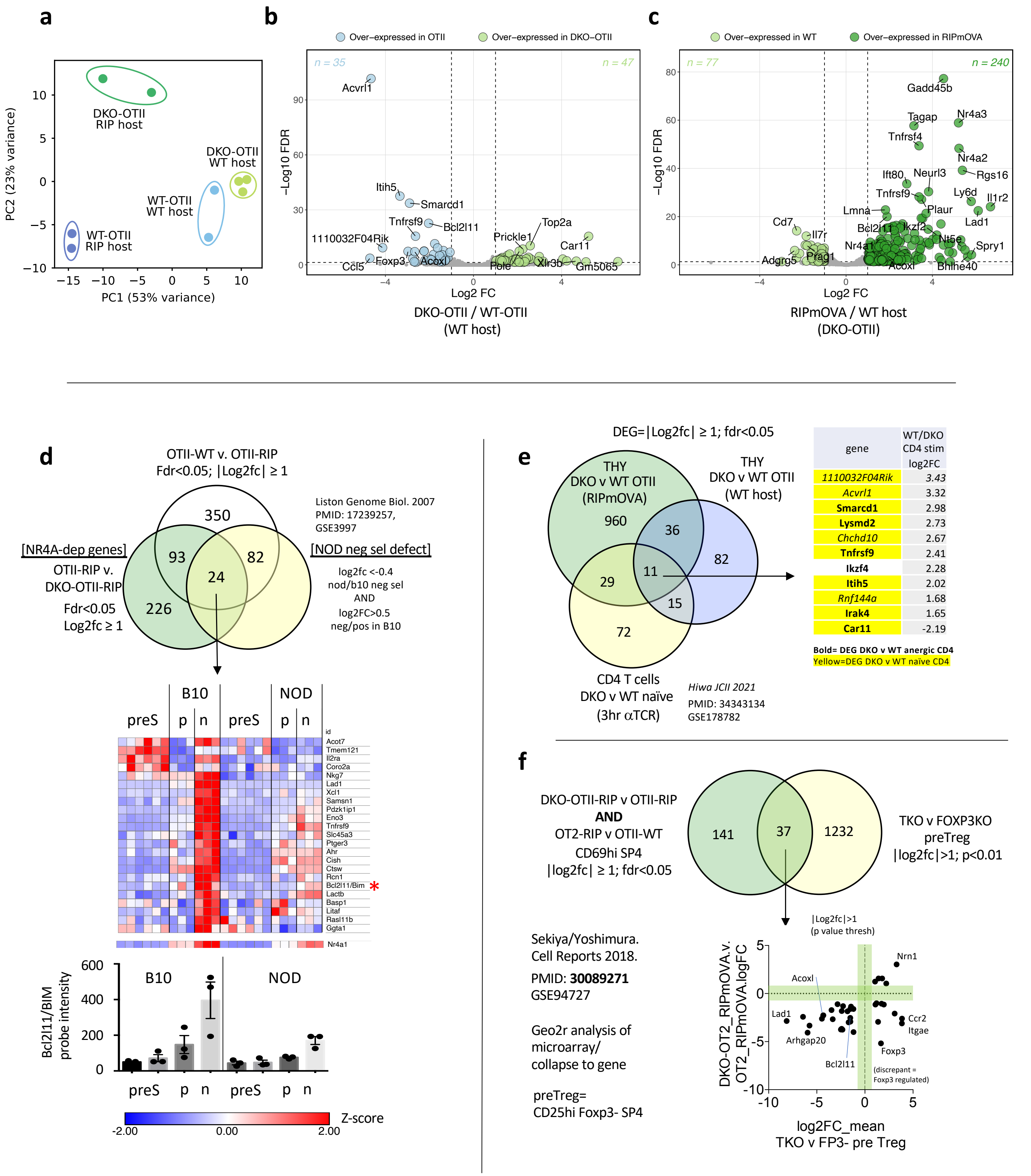

gene	WT/DKO CD4 stim log2FC
1110032F04Rik	*3.43*
Acvrl1	3.32
**Smarcd1**	2.98
**Lysmd2**	2.73
Chchd10	2.67
**Tnfrsf9**	2.41
**Ikzf4**	2.28
**Itih5**	2.02
Rnf144a	1.68
**Irak4**	1.65
**Car11**	-2.19

Supp Figure 6. Related to Main Figure 4

a. PCA plot depicts data from RNAseq experiment described in Main Fig 4a, GSE235101)

b. As in (a) except volcano plot depicts DEG (colored points) in WT-OTII vs. DKO-OTII thymocytes sorted from WT hosts.

c. As in (a) except volcano plot depicts DEG (colored points) DKO-OTII thymocytes sorted from either WT or RIPmOVA hosts.

d. Venn diagrams depict overlap between Nr4a-dependent genes and those induced by RIPmOVA in our RNAseq data set (white/green circle overlap) along with NOD-dependent genes induced in InsHEL model (yellow circle; PMID: 17239257, GSE3997). Filtering criteria as noted in figure. See also Supplementary Data 2a

e. Venn diagrams depict Nr4a-dependent genes in thymocytes from RIPmOVA and WT host chimeras (white, blue circles) compared to Nr4a-dependent genes from peripheral naïve (CD62Lhi CD25- CD73- FR4-) CD4 T cells (DKO v WT) stimulated through TCR for 3 hr (yellow circle, PMID: 34343134; GSE178782). Filtering criteria as noted in figure : =$|Log2fc| \geq 1$; $fdr<0.05$. see also Supplementary Data 2g which additionally identifies shared Nr4a-dependent genes among naïve CD4 ex vivo and among anergic CD4 (CD44hi CD62Llo CD25- CD73hi FR4hi) of same data set. Overlap among all data sets annotated with bold, yellow highlighting.

f. Venn diagram depicts Nr4a-regulated genes induced in RIPmOVA hosts in our data set (white circle) with Nr4a-dependent genes in pre-Treg (CD25hi Foxp3- thymocytes) defined by comparing Nr4ra1/2/3 TKO with Foxp3KO (PMID: 30089271; GSE94727 microarray data set analyzed via Geo2r and collapsed to gene). Filtering criteria as noted in figure. FC FC plot depicts overlap genes to identify concordantly Nr4a-dependent genes in bottom left quadrant. See also Supplementary Data 2f

Supp Figure 7. Transcriptional program induced by Nr4a family in thymus and periphery (related to Fig 4)

a

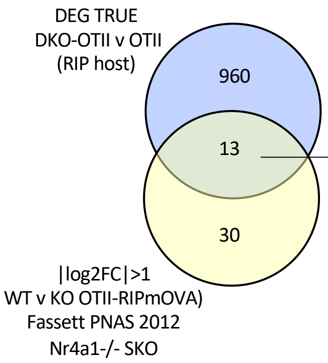

gene	Fassett PNAS Log2FC KO/WT (RIPmOVA)	This dataset Log2FC DKO/WT (RIPmOVA)
Tnfrsf9	-2.0057824	-4.401
Lactb	-1.6943213	-3.706
Lad1	-1.5395195	-3
Gsta4	-1.5311561	-2.215
Cyth3	-1.4579896	-2.851
Colq	-1.3327891	-2.134
Ggta1	-1.2515388	-1.932
Gsto1	-1.2075611	-1.822
Gnpnat1	-1.1713684	-1.669
Itih5	-1.1046974	-1.597
Hsdl2	-1.0831412	-1.308
Fdx1	-1.0409718	-1.22
Lysmd2	-1.0320936	-1.112

below threshold: Bcl2l11, Eno3

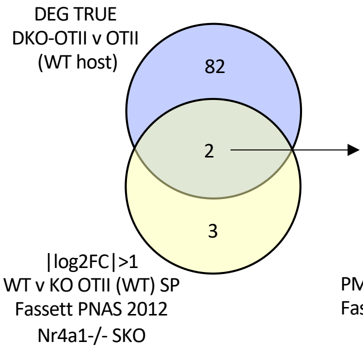

gene	Fassett PNAS Log2FC KO/WT (WT)	This dataset Log2FC DKO/WT (WT)
Acvrl1	-2.1520031	-4.653
Itih5	-1.3733272	-3.342

PMID: 22345564
Fassett/Mathis. PNAS 2012

---

b

Nr4a-dependent
log2FC < -2, n=23

SYMBOL	log2FC DKO v WT in RIP
Foxp3	-5.185
Tff1	-4.87
Itih5	-4.401
Nrgn	-3.974
Eno3	-3.748
Tnfrsf9	-3.706
Ccr6	-3.687
Tmem121	-3.671
C1ql3	-3.328
Gsto1	-3
Chchd10	-2.921
Lad1	-2.851
Lgals3	-2.694
Rasl11b	-2.666
Lrrc32	-2.646
Ahr	-2.544
Gzma	-2.537
Coro2a	-2.505
Il18rap	-2.489
Pla2g4f	-2.417
Ptger3	-2.267
Gsta4	-2.134
Sdc4	-2.077

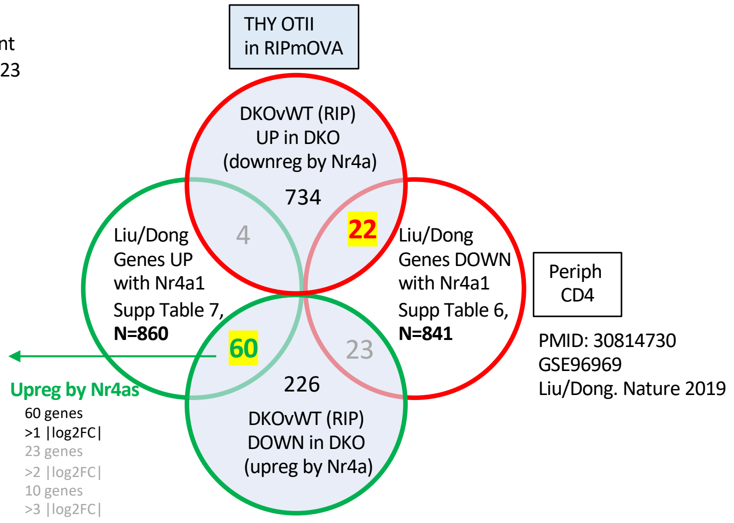

a. Venn diagrams compare DKO-dependent genes in either RIPmOVA (left) or WT (right) host chimeras with analogous samples from Nr4a1-/- OTII expressing RIPmOVA or not. Filtering criteria as noted in figure. Log2FC of overlap genes from both data sets as noted.

b. Venn diagrams compare DEG downregulated (top red/blue circle) or upregulated (bottom green/blue circle) between DKO-OTII and WT-OTII thymocytes from RIPmOVA host chimeras against Nr4a-dependent genes identified in peripheral T cells using Nr4a misexpression (white circles with red/green outlines; PMID: 30814730; GSE96969; filtering as applied by authors to generate Supplementary Data-Tables 6, 7 in reference). Yellow highlighting identifies concordantly Nr4a-regulated genes across data sets and table shows most highly Nr4a-upregulated shared genes. see also Supplementary Data 2h

Supp Figure 8. RTE exhibit imprint corresponding to thymic Ag encounter (related to Fig 5, 6)

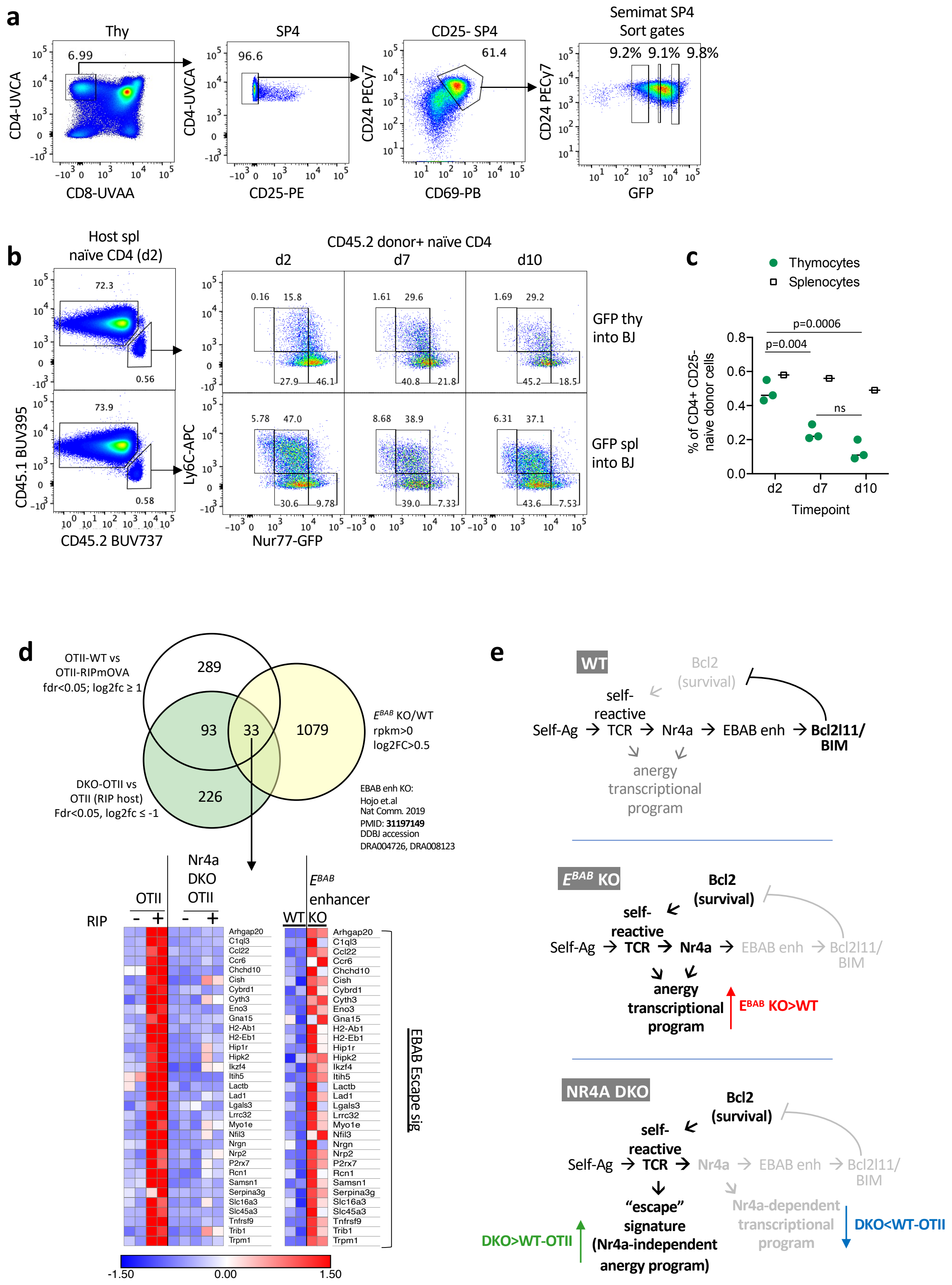

Supp Fig 8. Related to Main Fig 5 and 6.

a. Gating scheme for sort to identify semimature SP4 thymocytes (CD24hi CD69hi CD25-) with lo, med, hi GFP expression. Sorted cells subjected to RNAseq with data presented in Main Fig 5.

b. Gating scheme for adoptive transfer depicted in Main Fig 6e. CD45.2+ stain on recipient spleen identifies donor thymocytes (top) or splenocytes (bottom) retrieved from recipient CD45.1 host pre-gated to identify CD4+ CD62Lhi CD44- CD25- naïve cells. Righthand plots depict representative staining and gating for Nur77-eGFP and Ly6C to identify Pop A-D among CD45.2 donor cells in lefthand plots. Results are quantified in Main Fig 6f.

c. Graph depicts frequency of donor cells identified as in (b) at serial time points. N=3 biological replicates per time point for transferred thymocytes and N=1 reference per time point for transferred splenocytes.

d. Venn diagram depicts RIPmOVA-induced genes that are Nr4a dependent (white/green circle overlap) among genes overinduced in EBAB enhancer KO thymocytes (yellow circle). Heatmaps depict expression of those genes in our thymic RNAseq data set aligned against expression in WT or EBAB KO RNAseq data set (PMID: 31197149; see also Supplementary Data 4g)

e. models schematize regulation of Ag-dependent transcriptional modules in thymocytes that give an over-induced "escape" signature among either EBAB KO or Nr4a1/3 DKO cells that escape BIM-induced deletion. The escape signature is uncoupled from Nr4a-dependent program in DKO cells.

<u>Statistical tests:</u>

c, one way ANOVA with Tukey's multiple hypothesis test comparing thymocytes.

Source data are provided as a Source Data file.

Supp Figure 9. DKO thymocytes acquire an anergic program that persists in the periphery (related to Fig 7, 8)

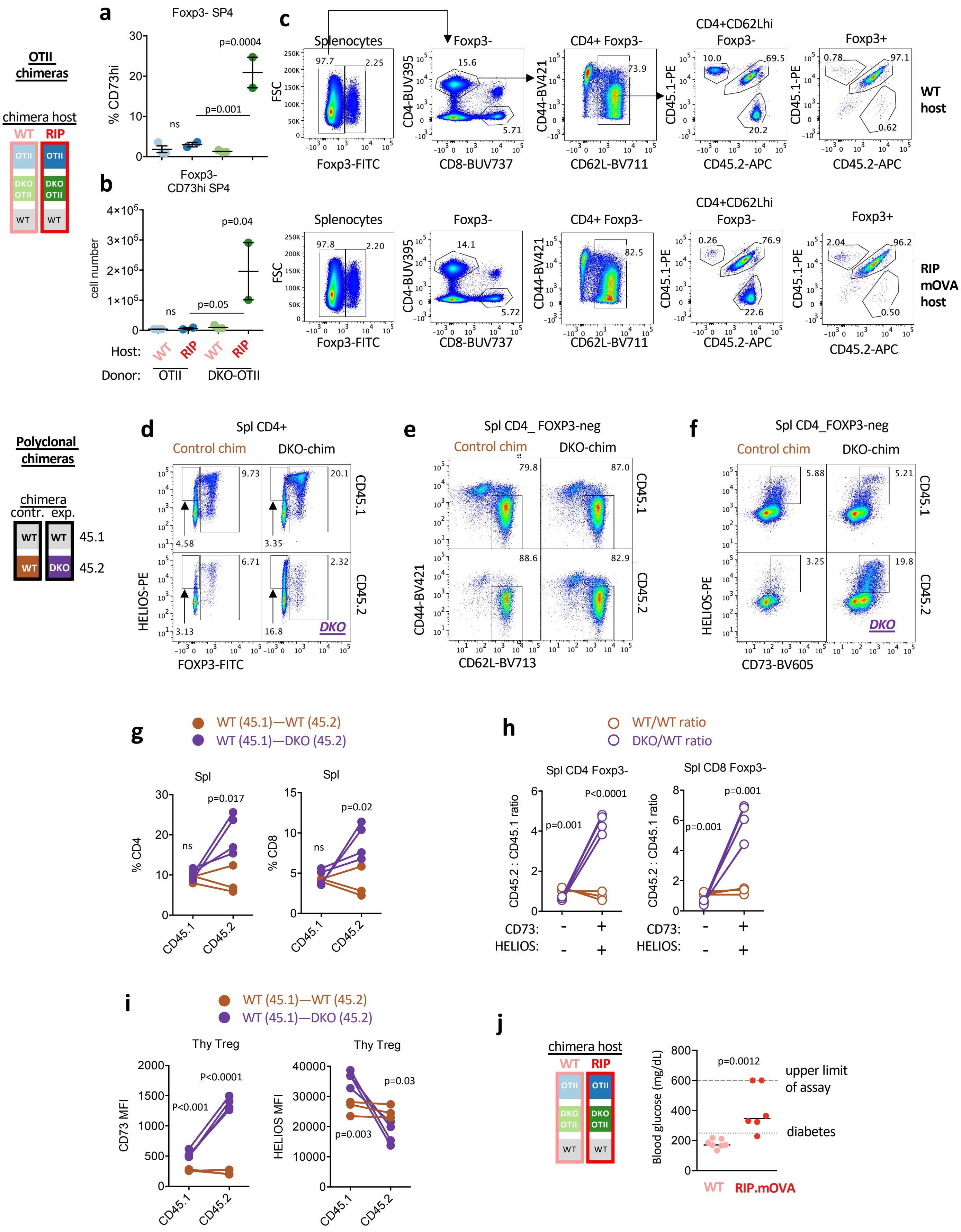

Supp Figure 9. Related to Main Figures 7, 8

a, b. Graphs depict quantification of CD73+ cells among SP4 Foxp3- OTII thymocytes of each genotype from RIPmOVA and WT host chimeras as gated in Fig 8h. Graphs depict N=3 WT host and N=2 RIPmOVA host chimeras +/- SEM.

c. Gating scheme depicts donor genotype contribution to Foxp3+ CD4 splenocytes and naïve Foxp3- CD4 splenocytes from RIPmOVA and WT host chimeras (see Fig 2a schematic).

d-f. Representative plots depict splenocytes from polyclonal chimeras (see Fig 1c schematic) stained to identify among each donor genotype intra-cellular FOXP3 and HELIOS within CD4+ gate (d), naïve CD62Lhi CD44lo FOXP3- CD4 T cells (e), and HELIOS+CD73+ gate among FOXP3- CD4 T cells (f).

g. Graphs depict % CD4 and % CD8 splenic T cells within each donor genotype from polyclonal chimeras (see Fig 1c schematic) as gated in Main Fig 9e. Lines connect donor cells from same chimera.

h. Graphs depict ratio of donor genotypes in polyclonal chimeras (Fig 1c schematic) from among CD73-HELIOS- and CD73+HELIOS+ FOXP3- splenic T cell populations (as gated in Supp Fig 9f above).

i. Graphs depict MFI of CD73 (left) and HELIOS (right) in thymic FOXP3+ SP4 Treg from each donor genotype in polyclonal chimeras (Fig 1c schematic). Lines connect donor cells from same chimera.

j. Blood glucose (mg/dL) from WT or RIPmOVA chimeras (see Main Fig 2a schematic) at 5.5-6.5 weeks following irradiation and bone marrow transfer. N=7 WT and N=6 RIPmOVA hosts (biological replicates).

<u>Statistical tests</u>:

a, b, one way ANOVA with Tukey's multiple hypothesis test.

g-j unpaired two-tailed parametric t-test, assume equal SD

Data in c represents N=2 DKO-OTII-RIPmOVA host chimeras and N=3 OTII-RIPmOVA host chimeras and reflect 3 independent sets of chimeras.

Data in d-f represent N=4 DKO:WT and N=3 control chimeras.

Source data are provided as a Source Data file.

Supp Figure 10. *Nr4a1* and *Nr4a3* are required for negative selection by tissue-specific Ag

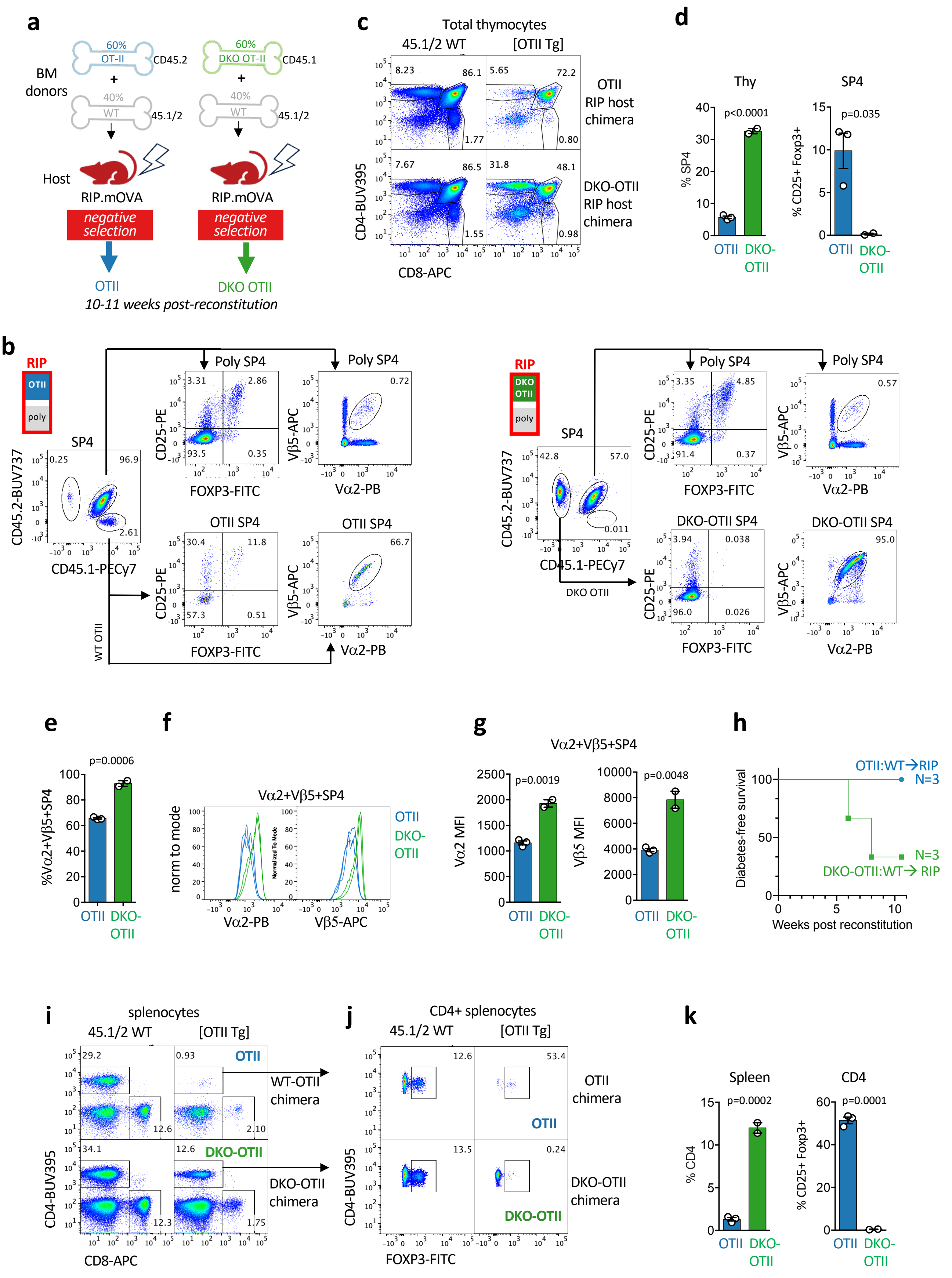

Supplementary Figure 10. Related to Main Figs 8k, l.

a. Schematic depicts radiation chimera design.

b-d. thymocytes from chimeras depicted in (a), harvested 10-11 weeks after BM transfer, and stained to detect congenic markers CD45.1/2, CD4/8 co-receptors, CD25, Vb5/Va2 TCR chains, and intra-cellular Foxp3 expression.

b. Gating scheme depicts donor genotypes (WT-OTII or DKO-OTII as well as admixed polyclonal donor) within SP4 compartment, and arrows direct to CD25/Foxp3 staining for each genotype to define Treg, pre-Treg, Tconv gates. Vα2 and Vβ5 staining identify OTII TCR.

c. Representative plots depict thymic subsets of each donor genotype

d. Quantification of subsets among each donor genotype as gated in b, c. values from N=2 DKO-OTII/RIPmOVA and N=3 WT-OTII/RIPmOVA chimeras +/-SEM.

e-g. Thymocytes from these RIPmOVA chimeras (a) were stained to detect vβ5 and vα2 TCR surface expression among subsets. Representative plots depict vβ5+ vα2+ OTII TCR gate among SP4 thymocytes of either WT-OTII or DKO-OTII donor genotype (e). Representative histograms depict surface expression of these TCR chains from SP4 thymocytes of either donor genotype and quantification of MFI +/- SEM (f, g).

h. Survival curve depicts time to onset of diabetic blood glucose of chimeras depicted in (a) for N=3 OTII:poly chimeras in RIPmOVA hosts and N=3 OTII:poly chimeras in RIPmOVA hosts.

i-k. splenocytes from chimeras depicted in (a), harvested and stained as in b-c. Representative plots depict peripheral splenic T cell subsets of each donor genotype (i), Treg gating among SP4/CD4 T cells of each donor genotype (j), and quantification of these values from N=2 DKO-OTII/RIPmOVA and N=3 WT-OTII/RIPmOVA chimeras +/- SEM (k).

<u>Statistical tests:</u>

d, e, g, k, unpaired two-tailed non-parametric t-tests

d, e, g, k graphs depict N=2 DKO-OTII/RIPmOVA and N=3 WT-OTII/RIPmOVA chimeras and are representative of at least 3 biological replicates of each.

Source data are provided as a Source Data file.

Supp Figure 11. Model

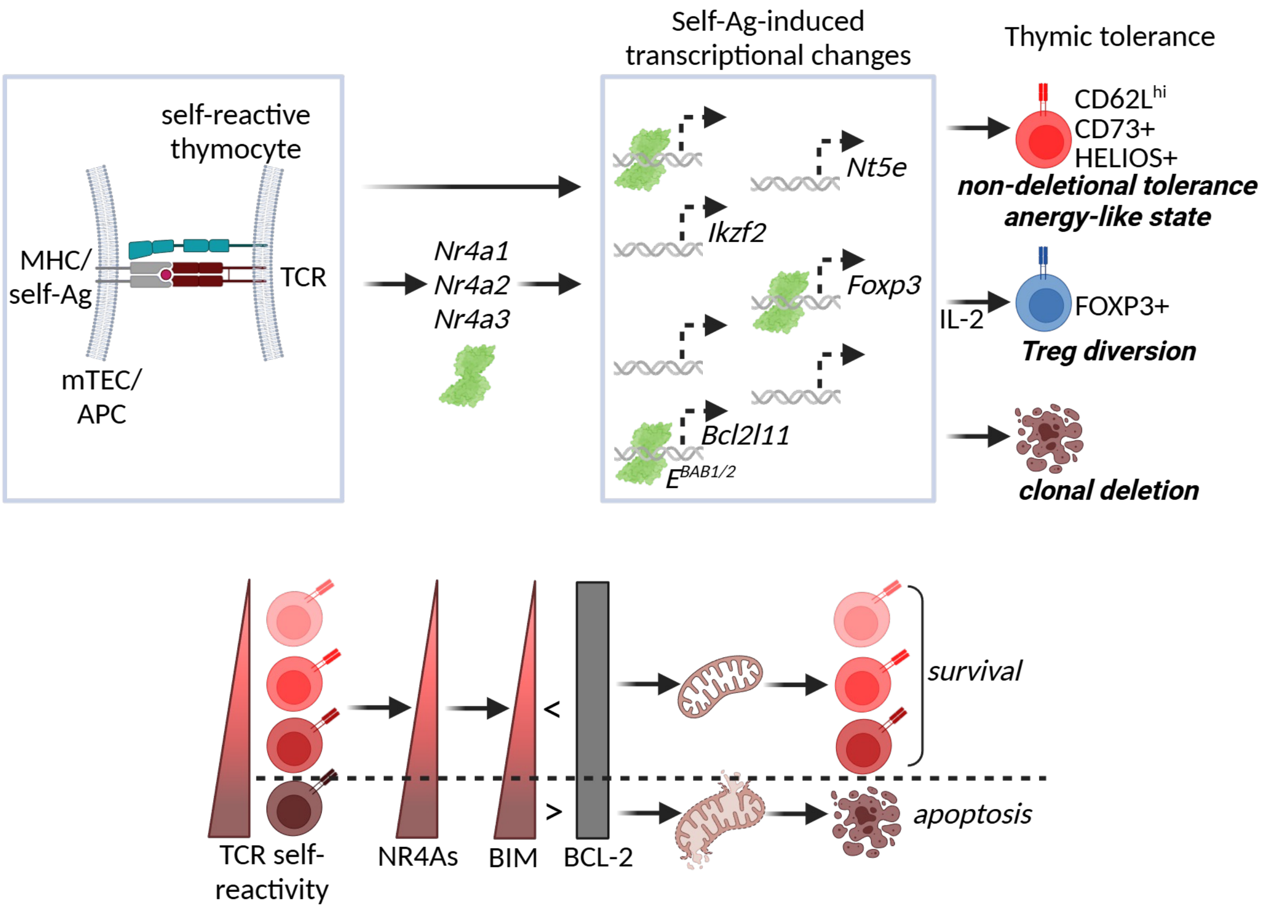

Nr4a genes are TCR-induced in SP thymocytes in the medulla during thymic selection where they induce both *Bcl2l11*/BIM expression via E^{BAB} enhancer engagement and a broader transcriptional program that promotes both Treg diversion and an anergy like program. We hypothesize that when BIM expression stoichiometrically overwhelms anti-apoptotic Bcl2 family members, highly signaled thymocytes with highest Nr4a expression trigger apoptosis. Created in BioRender. Nielsen, H. (2025) https://BioRender.com/v911322
