# Supplementary material for: Nr4a1 and Nr4a3 redundantly control clonal deletion and contribute to an anergy-like transcriptome in auto-reactive thymocytes to impose tolerance in mice
Source: Nat Commun. 2025 Jan 17;16:784. doi: 10.1038/s41467-025-55839-5 (PMC11742425; doi:10.1038/s41467-025-55839-5)
Supplement: Supplementary file 2 — Description of Additional Supplementary Files [file 41467_2025_55839_MOESM2_ESM.pdf]

Description of Additional Supplementary Files

Title: Transcriptional control of central T cell tolerance by NR4A family nuclear receptors

First author: Hailyn V. Nielsen

Corresponding authors: Byron B. Au-Yeung and Julie Zikherman

File name: Supplementary Data 1
Description: Tables 1a, b

File name: Supplementary Data 2
Description: Tables 2a-h

File name: Supplementary Data 3
Description: Tables 3a, b

File name: Supplementary Data 4
Description: Tables 4a-g

**Table**	**Title**
**1a**	DEG among CD69hi Va2Vb5 SP4: DKO OTII and WT OTII in WT or RIPmOVA hosts
**1b**	OCRs with Nr4a consensus sites within 200kb of TSS of Bcl2l11 (Immgen ATACseq)
**2a**	Comparison of OTII/RIPmOVA with InsHEL/3A9 model on B10 and NOD genetic backgrounds
**2b**	Comparison of OTII/RIPmOVA with HY-CD4 model of ubiquitous Ag-mediated thymic deletion
**2c**	Comparison of OTII/RIPmOVA with CITEseq negative selection clusters
**2d**	Comparison between CITEseq Treg and Neg selection clusters
**2e**	Comparison of DKO-OTII and WT-OTII/RIPmOVA data set with Nr4a1 SKO RIPmOVA data set
**2f**	Comparison of DKO-OTII and WT-OTII/RIPmOVA with TKO pre-Treg thymocytes
**2g**	Comparison of DKO-OTII and WT-OTII/RIPmOVA to DKO peripheral T cells
**2h**	Comparison of DKO-OTII and WT-OTII/RIPmOVA with Nr4a-dependent genes in CD4 T cells (overexpression)
**3a**	DEG between semimature SP4 thymocytes sorted according to GFP level from Nur77/Nr4a1-eGFP reporters
**3b**	Overlap of GFPhi SP4 transcriptomes from Nur77-eGFP reporter and OTII/RIPmOVA model
**4a**	Comparison of OTII/RIPmOVA gene set to peripheral naïve, stimulated, anergic CD4 T cells
**4b**	Comparison of OTII/RIPmOVA gene set to CD4 T cell tolerance (Ttol)
**4c**	Comparison of OTII/RIPmOVA gene set with CA-NFAT-RIT misexpression in CD4, CD8 T cells
**4d**	Comparison of OTII/RIPmOVA gene set with exhaustion-associated genes
**4e**	Overlap of Nur77-GFPhi SP4 transcriptome with DAR in naive CD4+ subsets sorted according to Nur77-GFP/Ly6C gates (pop D v A)
**4f**	Overlap of OTII/RIPmOVA SP4 transcriptome with DAR in naive CD4+ subsets sorted according to Nur77-GFP/Ly6C gates (pop D v A)
**4g**	Overlap of OTII/RIPmOVA gene set and Nr4a dependence of EBAB escape signature
