# Supplementary material for: Nr4a1 and Nr4a3 redundantly control clonal deletion and contribute to an anergy-like transcriptome in auto-reactive thymocytes to impose tolerance in mice
Source: Nat Commun. 2025 Jan 17;16:784. doi: 10.1038/s41467-025-55839-5 (PMC11742425; doi:10.1038/s41467-025-55839-5)
Supplement: Supplementary file 7 — Reporting Summary [file 41467_2025_55839_MOESM7_ESM.pdf]

nature portfolio

Corresponding author(s): Julie Zikherman : NCOMMS-24-38359B

Last updated by author(s): 12/13/2024

Reporting Summary

Nature Portfolio wishes to improve the reproducibility of the work that we publish. This form provides structure for consistency and transparency in reporting. For further information on Nature Portfolio policies, see our Editorial Policies and the Editorial Policy Checklist.

Statistics

For all statistical analyses, confirm that the following items are present in the figure legend, table legend, main text, or Methods section.

n/a	Confirmed	
☐	☒	The exact sample size (*n*) for each experimental group/condition, given as a discrete number and unit of measurement
☐	☒	A statement on whether measurements were taken from distinct samples or whether the same sample was measured repeatedly
☐	☒	The statistical test(s) used AND whether they are one- or two-sided<br>*Only common tests should be described solely by name; describe more complex techniques in the Methods section.*
☒	☐	A description of all covariates tested
☐	☒	A description of any assumptions or corrections, such as tests of normality and adjustment for multiple comparisons
☐	☒	A full description of the statistical parameters including central tendency (e.g. means) or other basic estimates (e.g. regression coefficient) AND variation (e.g. standard deviation) or associated estimates of uncertainty (e.g. confidence intervals)
☐	☒	For null hypothesis testing, the test statistic (e.g. *F*, *t*, *r*) with confidence intervals, effect sizes, degrees of freedom and *P* value noted<br>*Give P values as exact values whenever suitable.*
☒	☐	For Bayesian analysis, information on the choice of priors and Markov chain Monte Carlo settings
☒	☐	For hierarchical and complex designs, identification of the appropriate level for tests and full reporting of outcomes
☐	☒	Estimates of effect sizes (e.g. Cohen's *d*, Pearson's *r*), indicating how they were calculated

Our web collection on statistics for biologists contains articles on many of the points above.

Software and code

Policy information about availability of computer code

Data collection	Flow cytometry data were collected on a Fortessa and sorted on Aria (BD Biosciences).

Data analysis	Flow cytometry data was analyzed using FlowJo (v10) software (BD Biosciences). Statistical analysis and graphs were generated using Prism v6 and/or v10 (GraphPad Software, Inc).<br>For RNAseq data set (a), raw fastq files were mapped to the mm10 genome using STAR98 with the GENCODE vM17 reference transcriptome. PCR duplicate reads were marked with Picard MarkDuplicates and removed from downstream analyses. Reads mapping to exons for all unique ENTREZ genes were summarized using GenomicRanges99 in R v3.5.2 and normalized to reads per kilobase per million (RPKM). For data set (b), raw fastq files were adapter- and quality-trimmed with Trim Galore v0.6.10 and then mapped to the GRCm38 (mm10) genome using STAR v2.7.11b98 with the GENCODE vM25 gene annotation. PCR duplicate removal and Gene-level counts generation were handled by STAR with the options --bamRemoveDuplicatesType UniqueIdentical and --quantMode GeneCounts, respectively. Counts were normalized to transcripts per kilobase per million (TPM) Differentially expressed genes between sample groups within each of the two data sets were determined using DESeq2100, and genes that displayed an absolute log2 fold change ≥1= and Benjamini–Hochberg false discovery rate (FDR)–corrected p value ≤0.05 were considered significant. PCA plots were made using the plotPCA R function from gene-level counts normalized with variance stabilizing transformation100.<br>Heatmaps were generated using the Morpheus online tool (https://software.broadinstitute.org/morpheus/) with default clustering of rows (Pearson minus one) unless otherwise noted in figure legend. Volcano and FC plots were generated using R Statistical Software, Prism, or matplotlib.<br>Gene set enrichment analyses were performed with GSEA v4.3.3 (refs 97,98).<br>Public CITEseq data were analyzed using VISION graphical user interface (http://s133.cs.berkeley.edu:9002/Results.html), (GSE186078) (ref 38).

For manuscripts utilizing custom algorithms or software that are central to the research but not yet described in published literature, software must be made available to editors and reviewers. We strongly encourage code deposition in a community repository (e.g. GitHub). See the Nature Portfolio guidelines for submitting code & software for further information.

Data

Policy information about availability of data

All manuscripts must include a data availability statement. This statement should provide the following information, where applicable:

- Accession codes, unique identifiers, or web links for publicly available datasets
- A description of any restrictions on data availability
- For clinical datasets or third party data, please ensure that the statement adheres to our policy

Raw Fastq and processed data files (either RPKM or TPM) for the original RNA-seq analyses have been deposited in the NCBI Gene Expression Omnibus and are publicly available under accession code (a) GSE235101 and (b) GSE279344, and are also provided in Tables 1a, 3a. All previously published/public data sets analyzed in this manuscript are referenced with PMID and data repository locations where available in Tables 1-4. All other data that support the findings of this study are available from the corresponding author upon request.

Human research participants

Policy information about studies involving human research participants and Sex and Gender in Research.

Reporting on sex and gender	*Use the terms sex (biological attribute) and gender (shaped by social and cultural circumstances) carefully in order to avoid confusing both terms. Indicate if findings apply to only one sex or gender; describe whether sex and gender were considered in study design whether sex and/or gender was determined based on self-reporting or assigned and methods used. Provide in the source data disaggregated sex and gender data where this information has been collected, and consent has been obtained for sharing of individual-level data; provide overall numbers in this Reporting Summary. Please state if this information has not been collected. Report sex- and gender-based analyses where performed, justify reasons for lack of sex- and gender-based analysis.*
Population characteristics	*Describe the covariate-relevant population characteristics of the human research participants (e.g. age, genotypic information, past and current diagnosis and treatment categories). If you filled out the behavioural & social sciences study design questions and have nothing to add here, write "See above."*
Recruitment	*Describe how participants were recruited. Outline any potential self-selection bias or other biases that may be present and how these are likely to impact results.*
Ethics oversight	*Identify the organization(s) that approved the study protocol.*

Note that full information on the approval of the study protocol must also be provided in the manuscript.

Field-specific reporting

Please select the one below that is the best fit for your research. If you are not sure, read the appropriate sections before making your selection.

☒ Life sciences ☐ Behavioural & social sciences ☐ Ecological, evolutionary & environmental sciences

For a reference copy of the document with all sections, see nature.com/documents/nr-reporting-summary-flat.pdf

Life sciences study design

All studies must disclose on these points even when the disclosure is negative.

Sample size	Sample size was determined based on previous studies of similar nature.
Data exclusions	No data were excluded
Replication	At least two independent experiments and typically three or more - as noted in figure legends - were performed and all experiments were reliably reproduced. The reproducibility was based on magnitude and consistency of measurable differences between samples.
Randomization	n/a. cells from all animals stimulated in parallel with comparator reagents in every experiment. no randomization of mice was necessary.
Blinding	Experiments were not performed in a blinded fashion. Blinding was not relevant to our study, since we are studying and comparing the property of known cell types and genoptypes across comparator stimuli using objective data measurements (flow staining e.g.).

Reporting for specific materials, systems and methods

We require information from authors about some types of materials, experimental systems and methods used in many studies. Here, indicate whether each material, system or method listed is relevant to your study. If you are not sure if a list item applies to your research, read the appropriate section before selecting a response.

Materials & experimental systems

n/a	Involved in the study	
☐	☒	Antibodies
☒	☐	Eukaryotic cell lines
☒	☐	Palaeontology and archaeology
☐	☒	Animals and other organisms
☒	☐	Clinical data
☒	☐	Dual use research of concern

Methods

n/a	Involved in the study	
☒	☐	ChIP-seq
☐	☒	Flow cytometry
☒	☐	MRI-based neuroimaging

Antibodies

Antibodies used

Antibodies:
Abs for intra-cellular staining: HELIOS Ab conjugated to PE (clone 22F6, cat. 563801, BD Biosciences), BIM (clone C34C5; cat. 2933S, Cell Signaling) Rabbit mAb, FOXP3 Ab conjugated to APC or FITC (clone FJK-16s, Invitrogen). Anti-pERK (Phospho-p44/42 MAPK (T202/Y204) (clone 197G2, Cell Signaling) Rabbit Ab. Donkey Anti-Rabbit IgG (H+L) conjugated to APC (Jackson ImmunoResearch).

Stimulatory Abs: Anti-CD3 (clone 2c11) (BioLegend). Anti-CD28 (BioLegend cat: 102102 – clone 37.51). Goat anti-Armenian Hamster antibody secondary cross-link (Jackson ImmunoResearch).
Abs for surface markers: Streptavidin (SA) and Abs to CD4 (RM4-5 or GK1.5), CD5 (53-7.3), CD8 (53-6.7), CD24 (M1/69), CD25 (PC61.5), CD62L (MEL-14), CD69 (H1.2F3), CD44 (IM7), CD45.1 (A20), CD45.2 (104), CD73 (TY/11.8), FR4(12A5), Ly-6C(AL-21), Va2 (B20.1), Vb5(MR9-4), and TCRb (H57-597) conjugated to biotin or fluorophores (Biolegend, eBiosciences, BD, or Tonbo)

Primary Abs used at 1:200 except Foxp3 and pErk each used at 1:100, and secondary for BIM, pErk staining Donkey anti-rabbit APC was used at 1:100.

Validation

Commercial available antibodies were used which were validated by the manufacturers on their official website. Antibodies and clones used are common and described in the literature.

Animals and other research organisms

Policy information about studies involving animals; ARRIVE guidelines recommended for reporting animal research, and Sex and Gender in Research

Laboratory animals

All mice were housed in a specific pathogen-free facility at UCSF or Emory University according to institutional and National Institutes of Health guidelines. C57BL/6 (CD45.2) and BoyJ (CD45.1) mice (both considered WT here) were initially purchased from The Jackson Laboratory (Strain #:000664) or Charles River Labs (Strain Code 564), respectively. Nur77-eGFP BAC Tg mice were previously described [ref 26]. RAG-GFP mice were previously described and characterized as marker of RTEs [refs 75,95]. OT-II TCR transgenic mice [96] and RIP.mOVA transgenic mice [54] were previously described. Nr4a3-deficient allele was generated in our laboratory on the C57BL/6 genetic background as previously described [ref 77]. Nr4a3-/-Nr4a1-/- (DKO) mice were previously described [21] and

	were bred to OT-II mice to create the OT-II Nr4a1-/-Nr4a3-/- strain. BIM-deficient B6.129S1-Bcl2l11tm1.1Ast/J line (Jackson Strain #:004525)[ref 10] were crossed to Nur77-GFP mice. Foxp3-RFP [ref 97] mice crossed to Nur77-GFP were previously described [ref 73]. All strains were fully backcrossed to C57BL/6 genetic background for at least 6 generations. RIP.mOVA CD45.1/2 and WT CD45.1/2 mice were F1 progeny from RIP.mOVA x BoyJ crosses. OTII was crossed with BoyJ to generate and maintain OTII CD45.1 line. Mice of both sexes were used for experiments between the ages of 3 and 10 weeks except for BM chimeras as described below. Experimental and control chimeras were co-housed following irradiation. All experiments were approved by Institutional Animal Care & Use Committee (IACUC). Standard housing conditions used time controlled lighting on standard 12:12 light:dark cycle; humidity between 30-70%; temperature 68-79 degrees F. Euthanasia was carried out using approved protocols : carbon dioxide inhalation followed by cervical dislocation.
Wild animals	n/a
Reporting on sex	mice of both sexes were used for all assays and no differences in thymic phenotypes were observed. Therefore, all data represents pooled results using mice of both sexes.
Field-collected samples	n/a
Ethics oversight	All mice were housed in a specific pathogen-free facility at UCSF according to University and National Institutes of Health guidelines.

Note that full information on the approval of the study protocol must also be provided in the manuscript.

Flow Cytometry

Plots

Confirm that:

- [x] The axis labels state the marker and fluorochrome used (e.g. CD4-FITC).
- [x] The axis scales are clearly visible. Include numbers along axes only for bottom left plot of group (a 'group' is an analysis of identical markers).
- [x] All plots are contour plots with outliers or pseudocolor plots.
- [x] A numerical value for number of cells or percentage (with statistics) is provided.

Methodology

Sample preparation	Mouse cells were taken from thymus, spleens or lymph nodes of euthanized animals constructed via radiation chimeras as described in methods. Organs were meshed on cell strainers. ACK lysis was performed to remove RBCs for spleen samples. Culture condition depended on experimental detail outlined in the methods section and in figure legends although majority of analyses were directly ex vivo.
Instrument	Flow cytometry data was collected on Dual LSRFortessa.
Software	Flow cytometry data was analyzed using FlowJo (v10) software (Treestar Inc.). Statistical analysis and graphs were generated using Prism v6 (GraphPad Software, Inc).
Cell population abundance	noted throughout in gating schemes supplied in main and supp figs.
Gating strategy	gating scheme exemplars are presented throughout in main and supp figs.

- [x] Tick this box to confirm that a figure exemplifying the gating strategy is provided in the Supplementary Information.
